# Bilateral Ureteric Embolisation as Definitive Urinary Diversion in a Complex Radiation‐Induced Fistulation

**DOI:** 10.1111/ans.70843

**Published:** 2026-07-03

**Authors:** Rachel Cockburn, Alice Nicol, Mark Paxton, Kylie Gallagher

**Affiliations:** ^1^ Department of Urology Queen Elizabeth II Jubilee Hospital, Metro South Health Brisbane Australia; ^2^ Faculty of Medicine University of Queensland Brisbane Australia

## 1 Case

A 65‐year‐old male with a history of prostate cancer treated with external beam radiotherapy in 2022 presented with complex pelvic fistulation, complicated by recurrent pelvic sepsis. Initial urinary diversion was achieved with bilateral percutaneous nephrostomies and laparoscopic defunctioning colostomy. The patient was planned for hyperbaric oxygen therapy prior to formal surgical diversion.

His clinical course was complicated by renal pelvic haemorrhage with rupture of the right renal pelvis and persistent urine leak into the retroperitoneum. This caused recurrent fistulating infections (Figure 1), repeated drainage and pubic symphysis osteomyelitis. These infective insults left him malnourished and unsuitable for hyperbaric oxygen therapy or any future surgical intervention.

**FIGURE 1:**
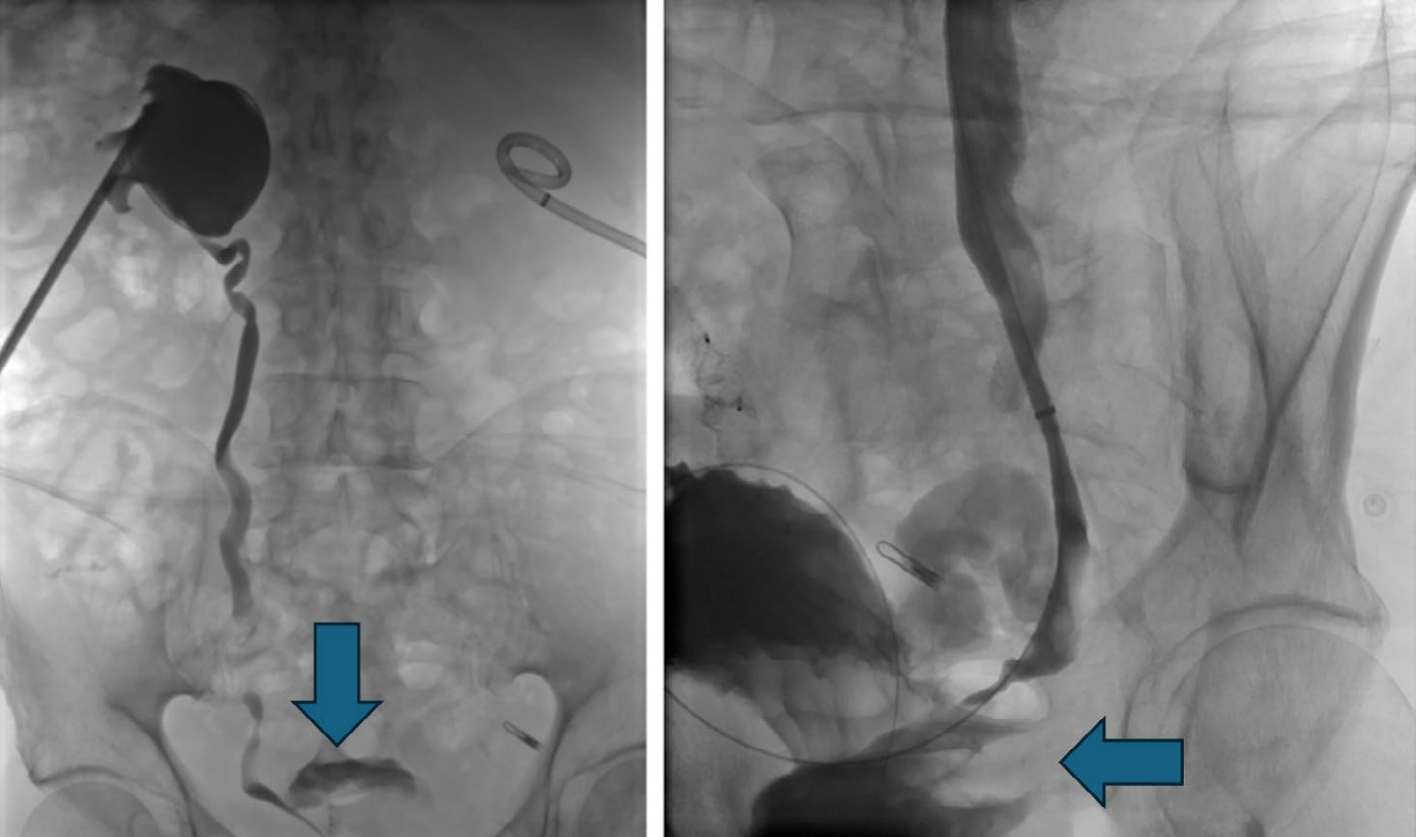
Preembolisation antegrade studies. The left image shows antegrade flow to the pelvis (see arrow). The right image demonstrates the urinary bladder fistulating with the prostate fossa inferiorly and the rectum posteriorly (see arrow).

In the context of persistent urinary leakage, definitive urinary diversion was pursued via interventional radiology by means of bilateral ureteric embolisation as a last resort.

Using existing nephrostomy access, the distal ureters were catheterised by a 45 cm 10Fr Flexor Ansel Guiding Sheath (Cook Medical; Figure 1). Two Amplatzer Vascular Plug type 4 (8 mm; Abbott) devices were deployed in the distal ureter. Subsequently, histoacryl–lipiodol (1:1 ratio; approximately 2 cc per ureter) was injected through a microcatheter jailed between two plugs for ureteric occlusion using a plug–glue–plug ‘sandwich’ technique (Figure 2). Completion contrast injection confirmed complete ureteric occlusion with no distal filling. The contralateral ureter was embolised using the same technique.

**FIGURE 2:**
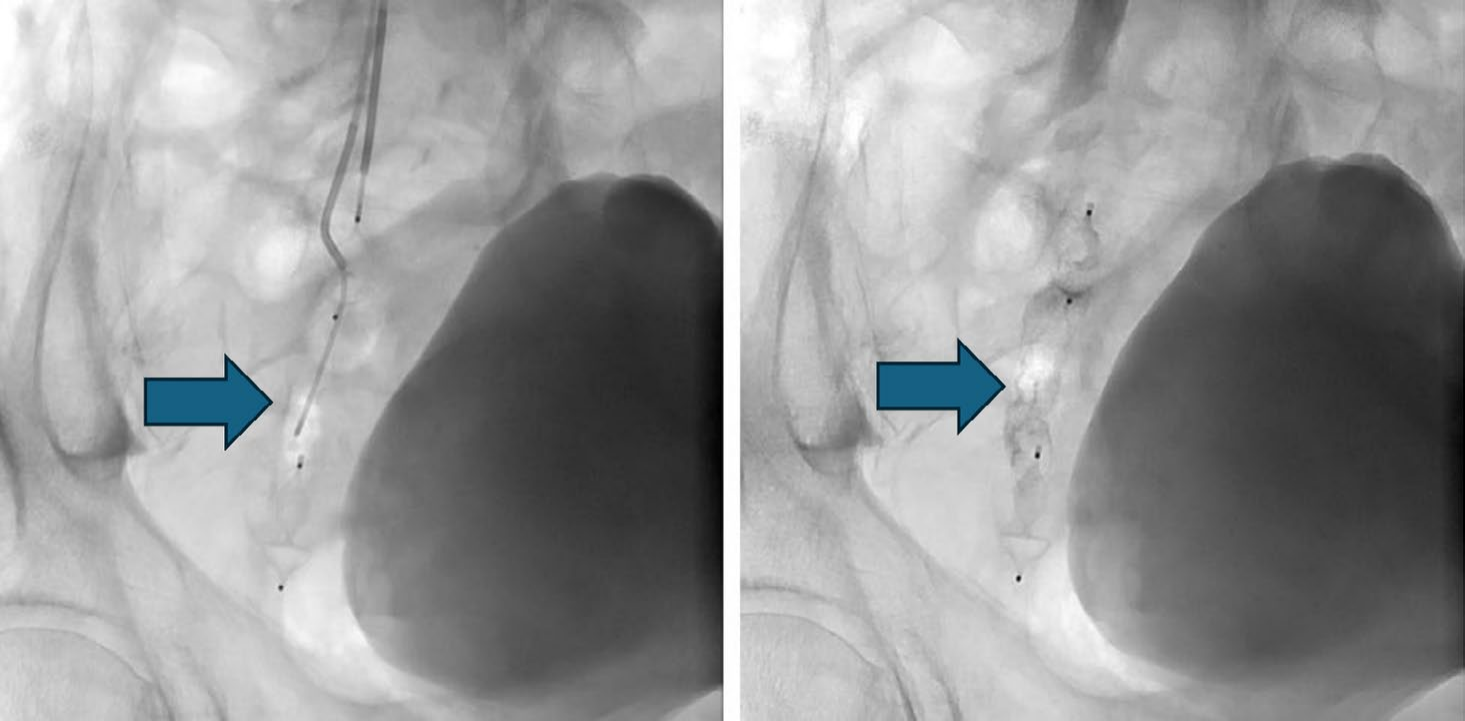
The left image demonstrates the microcatheter jailed between the two Amplatzer Vascular Plugs in the distal ureter prior to the injection of histoacryl‐lipiodol (see arrow). The right image demonstrates the final plug–glue–plug ‘sandwich’ configuration with a cast of occlusive glue between the plugs (see arrow).

Postprocedural imaging confirmed successful bilateral ureteric occlusion (Figure 3). The patient remains permanently nephrostomy‐dependent and has demonstrated sustained clinical improvement with resolution of urinary leakage and reduction in further fistulous complications at 2 years.

**FIGURE 3:**
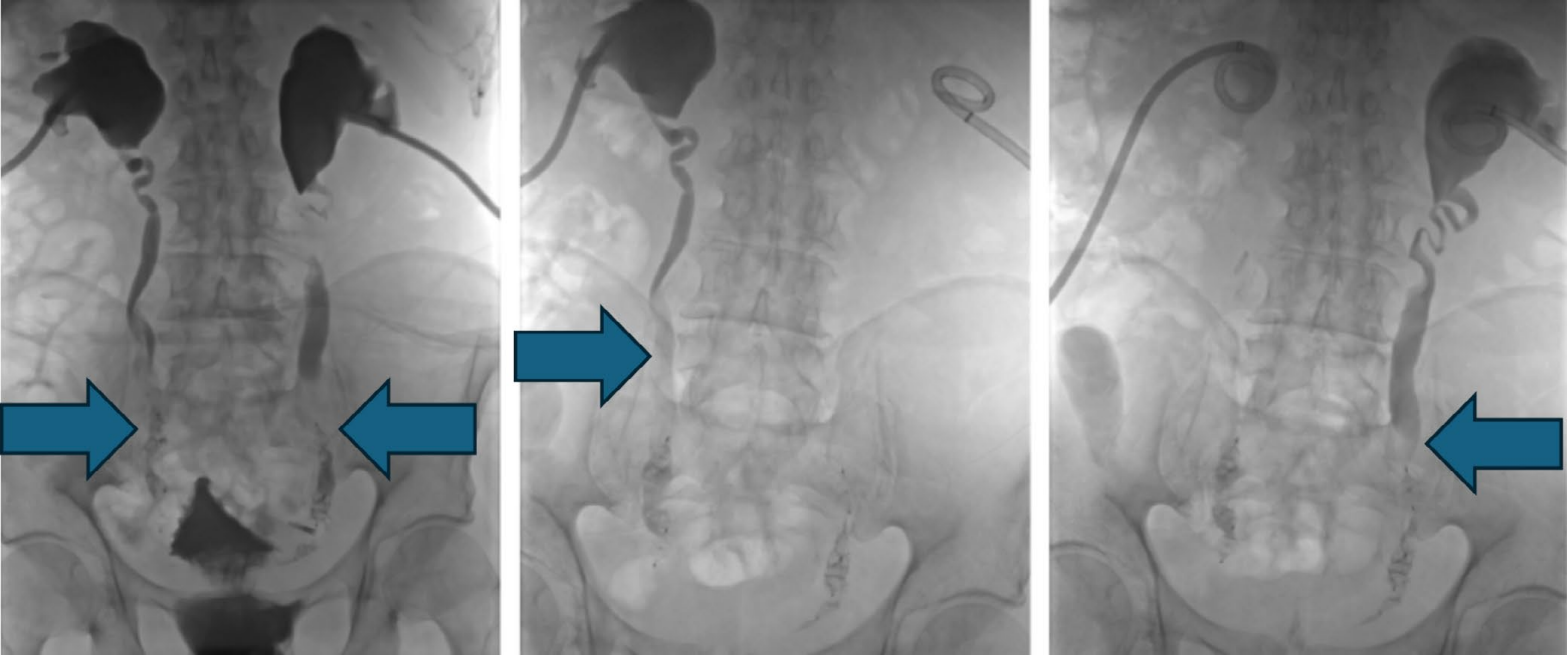
The left image is the final antegrade study upon completion of the procedure (see arrows for ureteric occlusion; note residual contrast in the bladder/pelvis prior to embolisation). The middle and right images are antegrade studies 2 months post procedure demonstrating distal ureteric occlusion (see arrows).

## 2 Discussion

Urinary diversion in the setting of radiation‐induced pelvic fistulation presents a significant management challenge. Prior radiotherapy, chronic infection and hostile pelvic anatomy frequently render reconstructive or extirpative surgery unsafe. Although nephrostomy diversion is commonly employed, it may fail to control distal urinary leakage, causing fistulae and sepsis.

Bilateral ureteric embolisation is an uncommon but underutilised method of definitive urinary diversion. The available literature consists largely of isolated case reports and small interventional radiology series, with varied indications including refractory haematuria from radiation cystitis, refluxing haematuria, ileal–ureteric anastomotic leaks and complex lower urinary tract fistulae [1, 2, 3].

Among these indications, fistula‐related pathology most closely resembles the present case. Series describing ureteric embolisation for vesicovaginal or enterovesicovaginal fistulae demonstrate that nephrostomy diversion alone is often insufficient, and that distal ureteric occlusion may be required to eliminate ongoing urinary leakage [1].

Various embolic materials have been described, including coils, glue and vascular plugs. Liquid embolic agents such as N‐butyl cyanoacrylate provide rapid occlusion but may recanalise over time, whereas mechanical devices such as Amplatzer plugs offer durability but lack immediate sealing [1, 4, 5].

The ‘sandwich technique’, in which liquid embolic is deployed between distal and proximal vascular plugs, combines the advantages of both modalities and has demonstrated high technical and clinical success in fistulous disease [1, 4, 5].

This case demonstrates successful use of this technique in a complex radiation‐injured pelvis complicated by osteomyelitis and extensive urinary extravasation in a patient unfit for surgery. Definitive elimination of distal urinary flow was critical to achieving infection control and sustained clinical stability.

Bilateral ureteric embolisation represents a rare but effective salvage option for urinary diversion in carefully selected patients when conventional surgical approaches are unfavourable. Multidisciplinary collaboration between urology and interventional radiology is essential in managing these challenging cases.

## Author Contributions

**Rachel Cockburn:** conceptualisation, investigation, writing – original draft, writing – review and editing. **Alice Nicol:** conceptualisation, writing – review and editing. **Mark Paxton:** conceptualisation, writing – review and editing. **Kylie Gallagher:** supervision, writing – review and editing.

## Funding

The authors have nothing to report.

## Consent

Written informed consent has been obtained from the patient to publish this paper.

## Conflicts of Interest

The authors declare no conflicts of interest.

## Data Availability

The data that support the findings of this study are available from the corresponding author upon reasonable request.
